# Analysis of growth and development levels and influencing factors in children aged 3–12 years in a certain region: a cross-sectional study

**DOI:** 10.3389/fpubh.2025.1523626

**Published:** 2025-09-10

**Authors:** Liping Gong, Yufeng Song, Shengquan Cheng, Jing Du, Juan Liang

**Affiliations:** Department of Pediatrics, Xijing Hospital, The Fourth Military Medical University, Xi’an, China

**Keywords:** child growth, development, cross-sectional study, influencing factors, bone body mass index, multi-center study

## Abstract

**Objective:**

This multi-center cross-sectional study aims to analyze growth and development levels and identify factors influencing these parameters among children aged 3–12 years in multiple regions of China.

**Methods:**

A total of 4,219 children (2,231 males and 1988 females) were included from local schools and community centers across 10 regions. Physical measurements (height, weight, and BMI) and bone age (assessed by R-series and C-series methods) were recorded. Parental heights were used to predict genetic adult height. A structured questionnaire provided data on demographics, family medical history, and lifestyle factors. Statistical analyses included t-tests, Pearson’s correlation, and multiple linear regression.

**Results:**

No significant sex differences were found in growth and development indices across age groups. Predicted adult height was higher in boys (176.17 ± 104.77 cm) than in girls (169.06 ± 7.13 cm). Age showed positive correlations with height (*r* = 0.400, *p <* 0.001), weight (*r* = 0.584, *p <* 0.001), and BMI (*r* = 0.699, *p <* 0.001). Father’s height was positively correlated with child height (*r* = 0.106, *p =* 0.041). Multiple linear regression indicated that age, weight, BMI, father’s height, and C-series bone age were significant predictors of child height (*p <* 0.001), with weight having the largest effect (*β* = 1.012). BMI and C-series bone age were significant predictors of weight (*p <* 0.001), while weight and height were significant predictors of BMI (*p <* 0.001).

**Conclusion:**

Growth and development in children are influenced by a combination of genetic, nutritional, and environmental factors. Understanding these influences can aid in developing targeted interventions to promote healthy growth patterns among children across diverse regions.

## 1 Introduction

Childhood is a critical period where substantial physical growth and cognitive development occur, setting the foundation for future health and well-being (1). The assessment of growth and development during these formative years is essential to ensure that children meet their potential and to identify any deviations from expected growth patterns that could signal underlying health issues (2). Globally, there is a diverse range of growth patterns due to differences in genetics, nutrition, socioeconomic status, and health care access, emphasizing the need for multi-center studies to understand specific growth trends and factors influencing development in children (3–5).

In recent years, with the acceleration of urbanization and changes in lifestyle, there has been a growing concern about the impact of these factors on the physical development of children (6–8). The prevalence of overweight and obesity in children is rising in many regions, posing a risk for various non-communicable diseases later in life (9). Simultaneously, in some areas, undernutrition remains a significant challenge, leading to stunted growth and development (10). These contrasting nutritional issues highlight the importance of analyzing growth indicators such as height, weight, and body mass index (BMI) to formulate appropriate public health strategies.

Childhood development is influenced by various environmental and genetic factors, which can affect growth velocity, the timing of puberty, and ultimately, adult stature (11–13). Factors such as parental height, socioeconomic status, and lifestyle choices, including diet and physical activity, play crucial roles in determining a child’s growth trajectory (14–16). Understanding these influences is critical to developing interventions that promote healthy growth and development and to addressing potential disparities within different population groups (17). This multi-center cross-sectional study aims to analyze the levels of growth and development and the factors influencing them among children aged 3–12 years across various geographical locations, providing valuable insights into the unique growth patterns and developmental challenges faced by these diverse populations.

## 2 Materials and methods

### 2.1 Study design and participants

This multi-center cross-sectional study was conducted across multiple regions to analyze the growth and development levels and influencing factors among children aged 3–12 years. A total of 4,219 children (2,231 males and 1988 females) were recruited from local schools and community centers across all participating regions. These regions comprised 10 distinct geographical locations across China: Beijing, Shanghai, Guangdong, Sichuan, Hubei, Shaanxi, Jilin, Gansu, Yunnan, Shandong These locations were strategically selected to represent a diverse spectrum of socioeconomic development and urban–rural settings, ensuring a broad representation of the diverse socio-demographic landscapes and living environments faced by children within the country. The inclusion criteria for participants were: age between 3 to 12 years, residence within the selected regions, and consent from parents or guardians. Children with chronic health conditions affecting growth, such as hormonal disorders or genetic syndromes, were excluded.

### 2.2 Data collection

Baseline characteristics were collected using a structured questionnaire administered to parents or guardians. The questionnaire included items on demographics, family medical history, and lifestyle factors. Physical development characteristics were assessed by measuring height, weight, and BMI. Growth percentiles were determined based on standard pediatric growth charts. Bone age assessment is a crucial tool in pediatric endocrinology and growth monitoring, providing insights into skeletal maturity that can inform predictions of adult height and identify potential growth disorders. These two methods are widely recognized for their distinct approaches to skeletal maturity assessment: the R-series method (based on the Tanner-Whitehouse 2 method) primarily evaluates the overall ossification patterns of the entire hand and wrist, providing a comprehensive assessment of skeletal maturation (18, 19). The C-series method (Tanner-Whitehouse 3 Carpal method), in contrast, focuses specifically on the maturity of the carpal bones (20). The combined use of these methods provides a more comprehensive and nuanced assessment of skeletal maturation, as they capture different aspects of bone development. Both R-series and C-series bone age assessment methods have demonstrated high reliability and validity in diverse populations globally. While their application and normative data for the general Chinese child population have been established in various studies, ongoing research continues to refine their specific validation in diverse regional cohorts within China (21). Our selection of these two methods was based on their established utility in clinical and research settings for assessing developmental progress and their ability to capture distinct yet complementary aspects of bone maturation.

Developmental status was categorized as advanced, normal, or delayed based on a combination of physical measurements and developmental milestones appropriate for the children’s age. Specifically, developmental status was determined by comparing individual child measurements and observed developmental milestones against age- and sex-specific growth charts and established developmental milestones recommended by WHO Child Growth Standards (22). ‘Advanced’ status was assigned when measurements or milestones were consistently above the 90th percentile or demonstrated early achievement of age-appropriate milestones. ‘Delayed’ status was assigned for measurements consistently below the 10th percentile or significant delays in achieving age-appropriate milestones. ‘Normal’ status encompassed children falling within the typical range (10th-90th percentile) for their age and sex and exhibiting expected developmental progress. Genetic adult height was predicted based on parental heights using established formulas.

### 2.3 Statistical analysis

Data were analyzed using SPSS version 26.0 (IBM Corp., Armonk, NY, United States). Descriptive statistics were used to summarize the baseline characteristics of the study population. Continuous variables were presented as mean ± standard deviation (SD), and categorical variables were presented as frequencies and percentages. Independent-samples t-tests were used to compare differences in continuous variables between two groups, while one-way analysis of variance (ANOVA) was used for comparisons among three or more groups. Pearson’s correlation coefficient (r) was used to assess the linear relationship between two continuous variables. Multiple linear regression analysis was performed to identify the factors influencing children’s height, weight, and BMI. All statistical tests were two-sided, and *p* < 0.05 was considered statistically significant.

### 2.4 Ethical considerations

The study protocol was reviewed and approved by the ethics committee of the local health authority. Informed consent was obtained from all parents or guardians before participation. The study was performed in accordance with the ethical standards of the Declaration of Helsinki.

## 3 Results

### 3.1 Sex differences in physical development characteristics of children aged 3–12 years

This study included 4,219 children, with 2,231 (52.9%) males and 1988 (47.1%) females. The age of the participants ranged from 3 to 12 years, with the majority falling within the 3–6 (36.5%) and 7–9 (36.9%) age groups. The predicted adult height based on genetics was 176.17 ± 10.48 cm for boys and 169.06 ± 7.13 cm for girls. Based on developmental status classification, 565 (25.3%) boys and 514 (25.9%) girls were categorized as advanced, 1,364 (61.1%) boys and 1,151 (57.9%) girls as normal, and 302 (13.6%) boys and 323 (16.3%) girls as delayed (Table 1).

**Table 1:** Baseline characteristics of the study population.

Characteristic	Male (*n* = 2,231)	Female (*n* = 1988)	Total (*n* = 4,219)
Age (years)			
3–6	924 (41.4%)	614 (30.9%)	1,538 (36.5%)
7–9	780 (35.0%)	776 (39.0%)	1,556 (36.9%)
10–12	527 (23.6%)	598 (30.1%)	1,125 (26.7%)
Height (cm)			
<120	709 (31.8%)	614 (30.9%)	1,323 (31.4%)
120 ≤ Height<150	1,323 (59.3%)	1,188 (59.8%)	2,511 (59.5%)
≥150	200 (8.9%)	186 (9.4%)	386 (9.1%)
Weight (kg)			
<20	628 (28.1%)	463 (23.3%)	1,091 (25.9%)
20 ≤ Weight<50	1,452 (65.1%)	1,381 (69.5%)	2,833 (67.2%)
≥50	151 (6.8%)	144 (7.2%)	295 (7.0%)
BMI (kg/m^2^)			
<18.5	1,699 (76.2%)	1,445 (72.7%)	3,144 (74.5%)
18.5 ≤ BMI < 24	412 (18.5%)	453 (22.8%)	865 (20.5%)
≥24	120 (5.4%)	90 (4.5%)	210 (5.0%)
Height percentile			
<10th	381 (17.1%)	360 (18.1%)	741 (17.6%)
10th-25th	412 (18.5%)	412 (20.7%)	824 (19.5%)
25th-50th	546 (24.5%)	422 (21.2%)	968 (23.0%)
50th-75th	463 (20.7%)	360 (18.1%)	823 (19.5%)
75th-90th	281 (12.6%)	198 (10.0%)	479 (11.4%)
>90th	148 (6.6%)	236 (11.9%)	384 (9.1%)
Weight percentile			
<10th	288 (12.9%)	268 (13.5%)	556 (13.2%)
10th-25th	442 (19.8%)	348 (17.5%)	790 (18.7%)
25th-50th	516 (23.1%)	429 (21.6%)	945 (22.4%)
50th-75th	360 (16.1%)	331 (16.7%)	691 (16.4%)
75th-90th	236 (10.6%)	198 (10.0%)	434 (10.3%)
>90th	389 (17.4%)	384 (19.3%)	773 (18.3%)
Genetic adult height (cm)	176.17 ± 10.48	169.06 ± 7.13	
R-series bone age (years)	8.23 ± 2.68	8.33 ± 2.66	
C-series bone age (years)	7.83 ± 2.44	8.05 ± 2.48	
Developmental status			
Advanced	565 (25.3%)	514 (25.9%)	1,079 (25.6%)
Normal	1,364 (61.1%)	1,151 (57.9%)	2,515 (59.6%)
Delayed	302 (13.6%)	323 (16.3%)	625 (14.8%)

### 3.2 Comparison of growth and development indices among children of different genders and age groups

**Figure 1:**
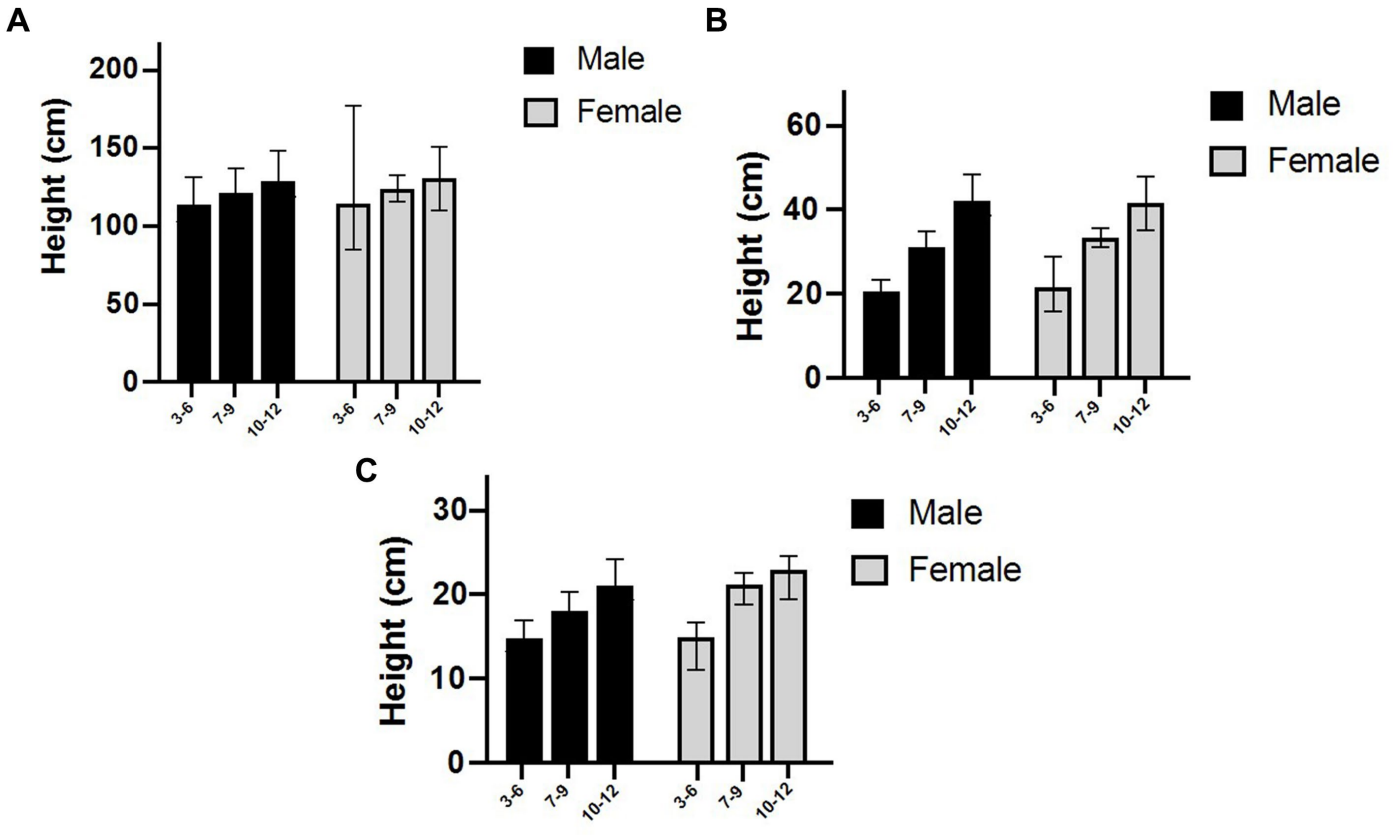
Comparison of mean height **(A)**, Weight **(B)**, and BMI **(C)** in children across different gender and age groups. **(A)** displays mean height (cm), **(B)** shows mean weight (kg), and **(C)** presents mean BMI (kg/m^2^). In all panels, male children are represented by black bars and female children by white bars, stratified by three age groups: 3–6 years, 7–9 years, and 10–12 years. This figure visually demonstrates the growth trends with increasing age for both sexes. Consistent with statistical findings (*p* > 0.05, as detailed in Table 2), no significant differences were observed in these growth and development indices between boys and girls within each respective age group.

There were no significant differences in height, weight, and BMI between boys and girls aged 3–6, 7–9, and 10–12 years (*p* > 0.05; Table 2; Figure 1).

**Table 2:** Comparison of growth and development indicators of children of different genders and age groups.

Indicator	Age group	Male	Female	*t*	*P*
Height (cm)	3–6	119.95 ± 13.87	121.12 ± 154.49	−0.482	0.630
	7–9	128.20 ± 17.66	128.05 ± 16.22	0.054	0.957
	10–12	138.621 ± 16.98	137.80 ± 14.10	0.271	0.787
Weight (kg)	3–6	21.31 ± 5.76	22.68 ± 12.35	−0.915	0.361
	7–9	29.27 ± 9.95	28.48 ± 9.66	0.495	0.621
	10–12	40.76 ± 14.21	38.96 ± 10.31	0.757	0.451
BMI (kg/m^2^)	3–6	14.53 ± 1.17	14.768 ± 2.78	−0.720	0.473
	7–9	17.22 ± 2.14	16.84 ± 2.02	1.141	0.255
	10–12	20.58 ± 4.11	20.08 ± 2.71	0.743	0.459

### 3.3 Comparison of height, weight, and BMI among children with different developmental statuses

No significant differences were found in height, weight, or BMI among children with different developmental status (*p* > 0.05). Although children with advanced development had slightly higher mean values for height, weight, and BMI compared to children with normal and delayed development, these differences were not statistically significant. Similarly, no significant differences were observed in height, weight, or BMI between children with delayed development and the other two groups (Table 3).

**Table 3:** Comparison of height, weight, and BMI of children with different developmental statuses.

Developmental status	Index	Male	Female	*t*	*P*
Advanced development	Height (cm)	129.01 ± 22.29	133.23 ± 19.50	−1.027	0.307
	Weight (kg)	33.47 ± 16.82	34.62 ± 16.24	−0.356	0.723
	BMI (kg/m^2^)	18.59 ± 3.48	18.41 ± 3.80	0.250	0.803
Normal development	Height (cm)	126.23 ± 16.39	125.71 ± 15.86	0.252	0.802
	Weight (kg)	27.08 ± 10.51	27.35 ± 10.82	−0.196	0.845
	BMI (kg/m^2^)	16.34 ± 2.87	16.60 ± 2.94	−0.707	0.481
Delayed development	Height (cm)	128.77 ± 11.94	132.34 ± 11.51	−1.167	0.248
	Weight (kg)	27.31 ± 7.57	30.13 ± 7.94	−1.397	0.168
	BMI (kg/m^2^)	16.41 ± 4.55	16.93 ± 2.67	−0.537	0.593

### 3.4 Correlation analysis of height, weight, and BMI among children with different age

**Figure 2:**
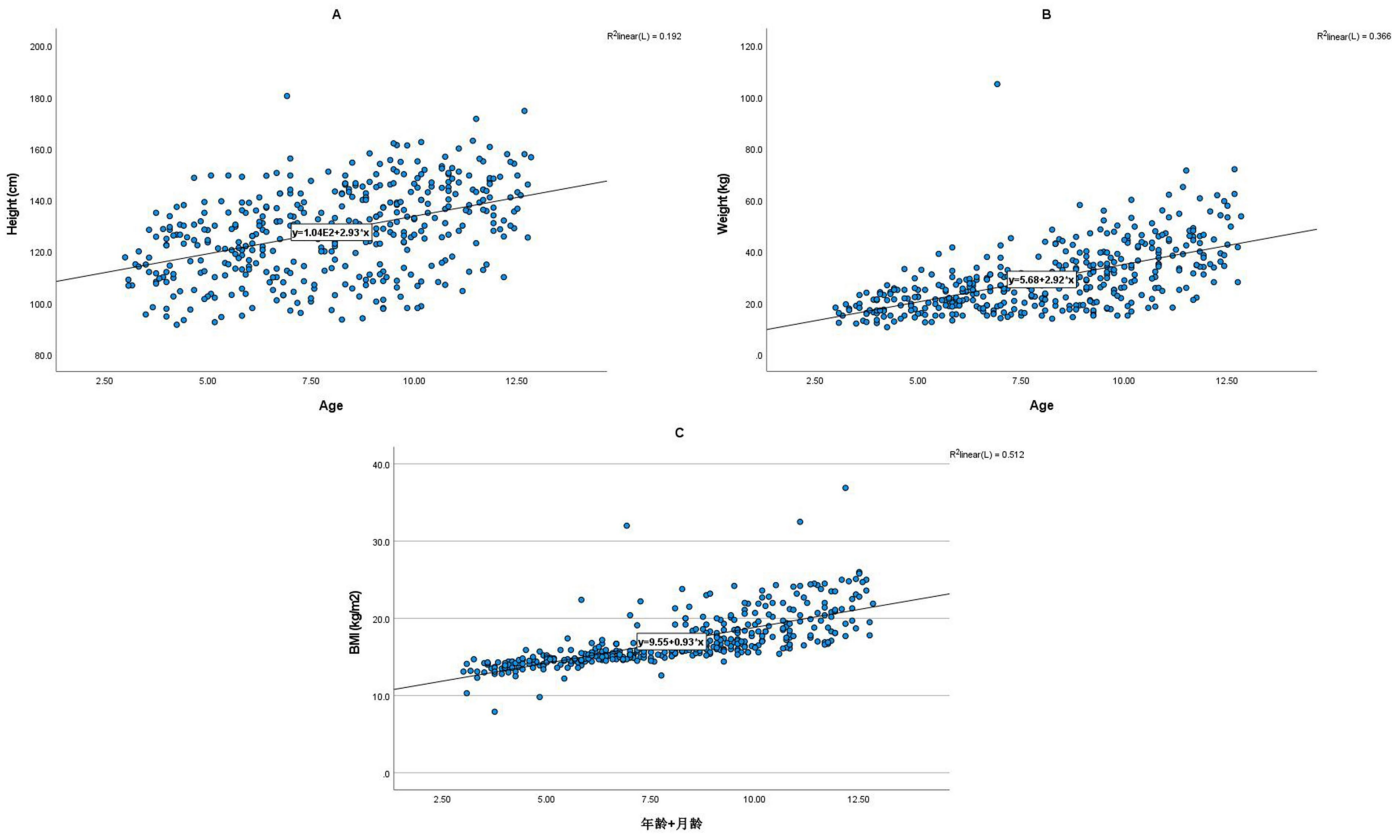
Linear relationships between age and children’s height **(A)**, Weight **(B)**, and BMI **(C)**. Each panel illustrates the positive linear correlation between age (in years, x-axis) and a specific anthropometric measure (y-axis). Panel **(A)** shows the relationship with Height (cm), Panel **(B)** with Weight (kg), and Panel **(C)** with BMI (kg/m^2^). The fitted regression lines demonstrate the strength of these relationships, with corresponding *R*^2^ values indicating the proportion of variance explained by age: Height (*R*^2^ = 0.192), Weight (*R*^2^ = 0.366), and BMI (*R*^2^ = 0.512). Age exhibited the strongest correlation with BMI, followed by weight, and then height.

Figure 2 shows the linear relationships between age and height, weight, and BMI. The results showed positive correlations between age and height, weight, and BMI. Among them, age had the strongest correlation with BMI (*R*^2^ = 0.512), followed by weight (*R*^2^ = 0.366), and the weakest correlation with height (*R*^2^ = 0.192).

### 3.5 Comparison of R-series and C-series bone age in children of different height levels and genders

There were no significant differences in R-series bone age and C-series bone age between boys and girls at different height levels (*p* > 0.05; Table 4).

**Table 4:** Comparison of R-series bone age and C-series bone age in children of different height levels and genders.

Height level	Bone age index	Male	Female	*t*	*p*
Short	R-series bone age	5.74 ± 3.16	6.43 ± 2.25	−0.496	0.628
	C-series bone age	6.00 ± 3.41	6.18 ± 2.36	−0.108	0.916
Lower middle	R-series bone age	7.25 ± 2.19	7.31 ± 2.22	−0.149	0.882
	C-series bone age	7.28 ± 2.21	7.34 ± 2.34	−0.152	0.880
Medium	R-series bone age	8.49 ± 2.71	8.94 ± 2.55	−1.106	0.270
	C-series bone age	7.97 ± 2.56	8.55 ± 2.36	−1.505	0.138
Upper middle	R-series bone age	9.16 ± 2.46	9.09 ± 2.70	0.107	0.915
	C-series bone age	8.25 ± 2.29	8.40 ± 2.53	−0.215	0.831
Tall	R-series bone age	10.38 ± 2.72	10.51 ± 3.85	−0.085	0.933
	C-series bone age	9.36 ± 1.43	9.68 ± 2.72	−0.320	0.754

### 3.6 Correlation analysis of different indicators with height, weight, and BMI

Age, R series bone age, and C series bone age were all significantly positively correlated with height, weight, and BMI (*p* < 0.001). Father’s height was significantly positively correlated with height (*p =* 0.041). There were no other significant correlations between the variables and height, weight, or BMI (Table 5).

**Table 5:** Correlation analysis results of different indicators with height, weight, and BMI.

Variable	Height		Weight		BMI	
	rs	*p*	rs	*p*	rs	*p*
Age	0.400	<0.001	0.584	<0.001	0.699	<0.001
Father’s height	0.106	0.041	0.082	0.113	0.031	0.546
Mother’s height	0.091	0.079	0.066	0.203	0.021	0.689
Genetic adult height	0.002	0.965	0.040	0.436	0.092	0.076
R-series bone age	0.906	<0.001	0.845	<0.001	0.564	<0.001
C-series bone age	0.919	<0.001	0.816	<0.001	0.532	<0.001

### 3.7 Multiple linear regression analysis of factors influencing children’s height, weight, and BMI

Multiple linear regression analysis showed that age, weight, BMI, father’s height and C series bone age were significant predictors of children’s height (*p <* 0.001). Among them, weight had the largest effect on height (*β* = 1.012), followed by C series bone age (*β* = 0.374) and BMI (*β* = −0.594). Age and father’s height had relatively small effects on height (*β* = 0.067 and β = 0.035). Gender, mother’s height, genetic adult height and R series bone age had no significant effects on height(*p >* 0.05; Table 6).

**Table 6:** Multiple linear regression analysis of factors influencing height.

Variable	Unstandardized coefficients		Standardized coefficients	*t*	*P*
	B	Standard error	Beta		
(Constant)	90.642	6.594		13.747	<0.001
Gender	−0.137	0.322	−0.004	−0.426	0.670
Age	0.408	0.088	0.067	4.631	<0.001
Weight (kg)	1.399	0.048	1.012	29.375	<0.001
BMI	−2.859	0.118	−0.594	−24.276	<0.001
Father’s height	0.101	0.030	0.035	3.387	0.001
Mother’s height	0.033	0.023	0.015	1.470	0.142
Genetic adult height	0.002	0.002	0.011	1.072	0.284
R-series bone age	0.052	0.212	0.008	0.246	0.806
C-series bone age	2.380	0.187	0.374	12.717	<0.001

BMI, height, C series bone age, R series bone age and age were significant predictors of children’s weight (*p <* 0.001). Among them, BMI had the greatest impact on weight (*β* = 0.575), followed by height (*β* = 0.695) and C series bone age (*β* = −0.212). R series bone age and age had relatively small effects on weight (*β* = 0.114 and −0.058). Gender, father’s height, mother’s height and genetic adult height had no statistically significant effect on weight(*p >* 0.05; Table 7).

**Table 7:** Multiple linear regression of factors influencing body weight.

Variable	Unstandardized coefficients		Standardized coefficients	*t*	*p*
	B	Standard error	Beta		
(Constant)	−62.085	3.623		−17.138	<0.001
Gender	0.230	0.193	0.010	1.195	0.233
Age	−0.256	0.053	−0.058	−4.861	<0.001
BMI	2.001	0.045	0.575	44.289	<0.001
Father’s height	−0.010	0.018	−0.005	−0.579	0.563
Mother’s height	−0.001	0.014	−0.001	−0.109	0.913
Genetic adult height	−0.001	0.001	−0.008	−0.911	0.363
R series bone age	0.536	0.124	0.114	4.323	<0.001
C series bone age	−0.977	0.125	−0.212	−7.838	<0.001
Height	0.502	0.017	0.695	29.375	<0.001

R series bone age, C series bone age, weight in kg, age, and height in cm were significant predictors of BMI in children (*p <* 0.001). Weight in kg had the largest effect on BMI (*β* = 1.466), followed by height in cm (*β* = −1.040) and C series bone age (*β* = 0.313). Age had a relatively smaller effect on BMI (*β* = 0.174). Sex, father’s height, mother’s height, and genetic adult height did not have a statistically significant effect on BMI (*p >* 0.05; Table 8).

**Table 8:** Multiple linear regression analysis of factors influencing BMI.

Variable	Unstandardized coefficients		Standardized coefficients	*t*	*p*
	B	Standard error	Beta		
(Constant)	26.634	1.744		15.271	<0.001
Sex	−0.132	0.088	−0.020	−1.491	0.137
Age	0.221	0.022	0.174	9.988	<0.001
Father’s height	0.010	0.008	0.017	1.249	0.213
Mother’s height	0.001	0.006	0.001	0.106	0.916
Genetic adult height	0.001	0.001	0.022	1.641	0.102
R series bone age	−0.134	0.058	−0.099	−2.311	0.021
C series bone age	0.415	0.058	0.313	7.166	<0.001
Height	−0.216	0.009	−1.040	−24.276	<0.001
Weight	0.421	0.010	1.466	44.289	<0.001

## 4 Discussion

In recent years, significant changes have been observed in the growth and development of children worldwide, closely related to socioeconomic development, improved nutritional status, and changes in lifestyle (23–25). Tian and Wang using data from the China Health and Nutrition Survey (CHNS), analyzed long-term trends in the growth and weight status of Chinese children from 1991 to 2011 (26). They found an increasing trend in standardized height, weight, BMI, and the prevalence of overweight and obesity. They also observed a widening disparity in overweight and obesity over time and pointed out the significant influence of family environment on children’s growth and weight status. Similarly, Mitsunaga and Yamauchi conducted a study on rural Zambian children and found that compared to the US population, Zambian children were shorter and had lower weight, but their nutritional status was not poor as judged by BMI (27). Additionally, children in the younger age groups were taller and heavier than 20 years ago, suggesting that improved socioeconomic conditions and healthcare standards have contributed to better growth and development in children.

Our study found no significant differences in growth and development indicators between boys and girls aged 3–12 years, which is consistent with the findings of Khadilkar et al. (28). Their study found no significant differences in the mean age- and sex-standardized z-scores for height, weight, BMI, and bone age between boys and girls of preschool and school-going age in India. However, in our study, the predicted adult height based on genetics was higher in boys than in girls, which may be related to genetic factors as well as differences in the timing and duration of puberty. Albertsson-Wikland et al. developed a novel type of BMI reference aligned with the age at onset of the pubertal growth spurt and found that stature at pubertal onset and childhood BMI can influence puberty-specific BMI gain (29). Additionally, our study found no statistically significant differences in height, weight, or BMI among children with different developmental statuses.

While children categorized with advanced development consistently showed slightly higher mean values for height, weight, and BMI compared to children with normal and delayed development, these numerical differences did not reach statistical significance. This may be related to the inclusion of diverse populations across centers and the criteria used to determine advanced and delayed development. Specifically, the categorization into ‘advanced,’ ‘normal,’ and ‘delayed’ developmental statuses in this study was based on a combination of physical measurements and broad developmental milestones. This general classification might not be sufficiently sensitive or granular to capture subtle variations in anthropometric measures across these groups. It is possible that children with developmental differences might exhibit non-physical manifestations (such as cognitive, social, emotional delays) that do not directly translate into significant differences in basic anthropometric indicators like height, weight, or BMI. Furthermore, the inherent variability within a large, diverse cross-sectional cohort could also contribute to masking statistically significant anthropometric differences between these broadly defined developmental groups. This non-significant finding suggests that relying solely on these basic physical measurements may not be comprehensive enough to fully reflect the nuances of developmental status in a diverse pediatric population, highlighting the need for more detailed and multidimensional developmental assessments in future studies.

Next, this study focuses on exploring the relationship between age and children’s growth and development indicators. Research has found that age was positively correlated with children’s height, weight, and BMI, with the strongest correlation observed between age and BMI. Choi et al. found that TMI (a new indirect measure of fat mass) was correlated with IGF-1 and IGFBP-3 levels in children and adolescents, and IGF-1 and IGFBP-3 levels are closely related to growth and development (30). Additionally, our study found no significant differences in R-series and C-series bone age between boys and girls at different height levels. This suggests that bone age may not be the sole factor influencing height, and other factors such as genetics, nutrition, and environment may also play significant roles. The study by Türkmen et al. also supports this view, as they found a significant correlation between lower extremity tendon thickness and age, sex, and anthropometric measurements (including height) in healthy children, but tendon thickness itself could not fully explain the height differences (31). Furthermore, our study found that age, R-series bone age, and C-series bone age were all significantly positively correlated with height, weight, and BMI, while father’s height was significantly positively correlated with height. This indicates that the influence of genetic factors on height should not be overlooked. The study by Aarestrup et al. also showed that birth weight, childhood BMI, and height were all associated with the risk of benign breast disease, further illustrating the impact of early growth and development on later health outcomes (32).

In order to further explore the factors affecting children’s growth and development, this study conducted in-depth analysis using multiple linear regression analysis method. The research results show that age is a significant predictor of height, weight, and BMI in children. This may be because, with increasing age, children’s bone development and muscle growth continue, leading to increases in height and weight, which in turn affect BMI. In addition, the study by Jensen et al. also pointed out that height in childhood is closely related to health status in adulthood, and the significant positive correlation between age and height found in our study also supports this view (33). In addition to age, genetic factors are also important factors affecting children’s growth and development. This study found that the father’s height was significantly positively correlated with the child’s height, which is consistent with the findings of Takagi et al., who found that the father’s height had a significant impact on the birth weight of Japanese children (34). This suggests that genetic factors play an important role in the growth and development of children. However, there was no significant correlation between the mother’s height and the child’s height in this study, which may be related to the variations across the included centers and other factors not included in the study.

It is worth noting that this study found that C-series bone age had a significant effect on height, weight, and BMI in children, while R-series bone age only had a significant effect on weight. The study by Meyer et al. also highlighted the importance of bone age in assessing children’s growth and development (35). C-series bone age and R-series bone age are two different bone age assessment methods, with C-series bone age mainly assessing the maturity of the carpal bones and R-series bone age assessing the maturity of the entire hand. The results of this study suggest that the maturity of the carpal bones may have a greater impact on children’s growth and development than the maturity of the entire hand. The results of this study indicate that children’s growth and development is a complex process influenced by a variety of factors. Age, sex, genetic factors, and bone age are all important factors affecting height, weight, and BMI in children. When assessing the growth and development of children, these factors should be considered comprehensively and individualized interventions should be developed to promote the healthy growth of children.

The insights gleaned from this study have crucial implications for the development of targeted child health policies and practices, particularly in China and other developing countries facing similar growth and developmental challenges. Understanding these influences can aid in developing targeted interventions to promote healthy growth patterns among children across diverse regions. Public health strategies should be tailored to address identified influencing factors, including genetic predispositions, nutritional needs, and environmental conditions that contribute to observed growth trends. Specifically, interventions should focus on strengthening routine growth monitoring programs by implementing standardized, regular monitoring of height, weight, and BMI from early childhood, utilizing established growth charts like WHO Child Growth Standards, to enable early detection of deviations from healthy growth patterns. Developing targeted nutritional interventions that promote balanced diets rich in essential nutrients, including adequate protein and vitamin D, through educational programs for parents and caregivers is also vital, given the significant influence of weight and BMI on height. Furthermore, encouraging regular physical activity through community- and school-based programs is crucial to support healthy bone development and weight management, which are essential for overall growth. Enhancing parental education on genetic influences, such as the impact of paternal height, and the importance of monitoring developmental milestones can foster realistic expectations and facilitate early intervention when needed. Improving the application of bone age assessments, particularly training healthcare professionals in the accurate and nuanced interpretation of the C-series method due to its stronger influence in our findings, can better predict growth potential and guide clinical decisions. Finally, policies should also address broader socioeconomic and environmental determinants, such as improving access to healthcare, sanitation, and education, as these overarching factors significantly impact child growth and development in diverse regions.

This study is a multi-center cross-sectional study, which can only reflect the growth and development status of the subjects at a certain point in time, and cannot accurately assess the growth rate and trend. In addition, this study only included indicators such as height, weight, and bone age, and did not collect other important variables that may significantly affect children’s growth and development. These include, but are not limited to, detailed nutritional status (e.g., specific food intake, protein consumption, vitamin D levels), physical activity levels, sleep duration, and psychosocial factors (e.g., family environment, socioeconomic status, and stress levels). The omission of these important confounding variables represents a limitation of the current cross-sectional study, potentially introducing bias and influencing the interpretation of our findings. For instance, children’s growth trajectories are known to be intricately linked with their nutritional intake and physical activity patterns. Failure to account for these factors may lead to an incomplete understanding of the observed anthropometric variations and could potentially overestimate or underestimate the direct influence of the studied variables (age, parental height, bone age) on height, weight, and BMI. Therefore, the observed associations, while statistically significant, should be interpreted with caution, acknowledging that unmeasured confounding factors could be at play. Dathan-Stumpf et al. pointed out that serum lipid concentrations and anthropometric parameters of children in the first year of life are affected by multiple factors, including maternal BMI, lipid levels, and socioeconomic status (36). Future research should prioritize incorporating a more comprehensive set of variables to gain a holistic understanding of the intricate interplay between genetic, nutritional, environmental, and psychosocial factors influencing child growth and development. For example, Zhao et al.’s study showed that adiposity is not beneficial to bone mineral density in 0-5-year-old Chinese children (37). Therefore, future research could consider including factors such as children’s dietary habits, exercise status, and parental obesity status in the analysis to gain a more comprehensive understanding of the factors influencing children’s growth and development. Such longitudinal and multi-faceted studies would provide more robust evidence for developing targeted and effective public health interventions aimed at promoting healthy growth patterns across diverse child populations.

## 5 Conclusion

This multi-center cross-sectional study provides valuable insights into the growth and development levels of children aged 3–12 years across different regions. Our findings suggest that no significant sex differences in growth and development indices across the considered age groups, with predicted adult height being higher in boys than in girls, potentially due to genetic factors and different puberty timelines. Age was positively correlated with height, weight, and BMI, with weight having the largest effect on predicted child height among the variables considered in the multiple linear regression analysis. Moreover, both R-series and C-series bone age assessments were significant predictors of child height, weight, and BMI, with C-series bone age showing a stronger influence. Paternal height also showed a significant correlation with child height, emphasizing the role of genetic factors. The lack of significant differences in physical development characteristics among children with different developmental statuses suggests that these classifications may require refinement for more accurate assessments. Moreover, the study underlines the importance of considering both genetic and environmental influences when evaluating child growth and development patterns. To promote healthy growth patterns among children, targeted interventions that consider these influencing factors are crucial. Public health strategies should be tailored to address genetic predispositions, nutritional needs, and environmental conditions that contribute to the observed growth trends. Continued research, including longitudinal studies, would be beneficial to understand the dynamic growth trajectories and the long-term impact of early growth patterns on adult health outcomes.

## Data Availability

The original contributions presented in the study are included in the article/supplementary material, further inquiries can be directed to the corresponding author/s.
